# Midcarpal tenodeses versus partial arthrodeses for stage II SLAC/SNAC wrists: Long-term outcomes from a single-surgeon comparative series

**DOI:** 10.1051/sicotj/2025069

**Published:** 2026-02-23

**Authors:** Spyridon Maris, Emmanouil Apergis, Alexandros Apostolopoulos, Dimitra Melissaridou, Panagiotis Koulouvaris, Panayiotis J. Papagelopoulos, Olga Savvidou

**Affiliations:** 1 Department of Orthopaedics and Traumatology, General Hospital of Athens Korgialeneio-Benakeio Hellenic Red Cross 11526 Athens Greece; 2 1st Department of Orthopaedic Surgery, Attikon University General Hospital 12462 Athens Greece

**Keywords:** SLAC II, SNAC II, Midcarpal tenodesis, Partial arthrodesis

## Abstract

*Background*: Stage II scapholunate advanced collapse (SLAC) and scaphoid nonunion advanced collapse (SNAC) are commonly treated with partial arthrodeses or motion-preserving techniques such as midcarpal tenodeses. Comparative evidence with long-term follow-up remains limited. *Purpose*: To compare long-term clinical and functional outcomes of midcarpal tenodeses and partial arthrodeses in patients with stage II SLAC/SNAC, by evaluating grip strength, range of motion, patient-reported outcomes, and reoperation rates. *Methods*: A retrospective review was performed on 21 patients operated by a single surgeon with a mean follow-up of 103 months. Nine underwent midcarpal tenodeses (FCR or ECRB based), and twelve underwent partial arthrodeses (four-corner fusion or capitolunate fusion). Outcomes included grip strength, range of motion, radiographs, and PROMs (VAS, DASH, PRWE, Mayo Wrist Score). *Results*: Both procedures produced comparable long-term outcomes. Mean postoperative grip strength was 27.9 kg (~75% of the contralateral side). PROMs were similar between groups (DASH 12.1, PRWE 15.5). Importantly, no complications, non-unions, or conversions to salvage arthrodesis occurred in either group during long-term follow-up. *Conclusion*: Midcarpal tenodeses and partial arthrodeses yield similarly durable outcomes in stage II SLAC/SNAC wrists. Tenodeses preserve motion and are suitable for patients with preserved cartilage, whereas partial arthrodeses offer predictable stability when midcarpal degeneration is present. Treatment should be individualized according to cartilage status, functional demands, and patient expectations.

## Introduction

Scapholunate advanced collapse (SLAC) and scaphoid nonunion advanced collapse (SNAC) represent the most common patterns of degenerative wrist arthritis, following a predictable progression from radioscaphoid degeneration to midcarpal involvement (stage II) and ultimately capitolunate joint destruction (stage III) [1, 2]. Stage II is particularly significant because midcarpal cartilage may still be preserved, allowing consideration of both motion-preserving and fusion-based strategies.

Several surgical options exist for stage II. Partial arthrodeses – including four-corner fusion (4CF), proximal row carpectomy (PRC), Three-corner fusion (3-CF), as well as capitolunate fusion (CLF) – offer durable pain relief with high union rates and consistent long-term outcomes [1, 3–5]. Conversely, motion-preserving soft-tissue reconstructions, such as FCR- and ECRB-based midcarpal tenodeses, may improve pain and preserve motion in patients with intact midcarpal cartilage, though durability remains a concern [6–10].

Despite widespread use of these procedures, comparative long-term evidence is limited. Most published studies evaluate each procedure independently, and direct comparisons between midcarpal tenodeses and partial arthrodeses for stage II SLAC/SNAC are almost nonexistent [3, 6, 7, 9, 11, 12].

Midcarpal tenodesis in stage II SLAC/SNAC is considered an appropriate option when the radiolunate and capitolunate cartilage are fully preserved, allowing a motion-preserving reconstruction without jeopardizing future salvage procedures. The procedure appeals to surgeons and patients wishing to avoid fusing intact joints or creating incongruent articulations, and it carries the expectation that a well-aligned capitolunate relationship can be maintained over time. For these reasons, midcarpal tenodesis is an attractive alternative to classic palliative procedures in carefully selected stage II wrists.

The purpose of this study was to compare long-term outcomes of midcarpal tenodeses versus partial arthrodeses in stage II SLAC/SNAC wrists by evaluating grip strength, range of motion, PROMs, radiographic progression, complications, and reoperations.

## Materials and methods

### Study design and patient selection

This retrospective comparative study included adults with stage II SLAC or SNAC wrist arthritis treated by a single surgeon (2002–2021). Inclusion criteria were: radiographic stage II disease, completion of either midcarpal tenodesis or partial arthrodesis, and ≥24 months of follow-up. Patients with inflammatory arthropathy, prior wrist fusion, or stage III collapse were excluded [3, 10].

A total of twenty-one patients met the inclusion criteria, with nine undergoing midcarpal tenodeses and twelve undergoing partial arthrodeses. The mean age was 52.8 ± 11.8 years, with no significant differences between groups. Stage II SNAC was more frequent in the arthrodesis group, whereas SLAC II was evenly distributed. The mean follow-up was 103.3 ± 65.5 months for the entire cohort, with no significant intergroup differences in follow-up duration, consistent with similarly structured SLAC/SNAC series [1, 3]. Patient characteristics are summarized in Table 1. All the patients underwent grip strength and Range of motion tests, radiographic evaluation, and PROMs (VAS, DASH, PRWE, Mayo Wrist Score).

**Table 1:** Sample’s characteristics.

		Total		Type of surgery				*P*
				Tenodeses		Arthrodeses		
		*Ν*	%	*Ν*	%	*Ν*	%	
Type of arthritis	SNAC II	12	57.1	3	33.3	9	75.0	0,087^++^
	SLAC II	9	42.9	6	66.7	3	25.0	
Age, mean (SD)		52.8 (11.8)		57.1 (14.1)		49.5 (8.9)		0.147^+^
Sex	Male	20	95.2	8	88.9	12	100.0	0,429^++^
	Female	1	4.8	1	11.1	0	0.0	
Dominant hand	Right	17	81.0	7	77.8	10	83.3	>0.999^++^
	Left	4	19.0	2	22.2	2	16.7	
Operated hand	Right	13	61.9	5	55.6	8	66.7	0.673^++^
	Left	8	38.1	4	44.4	4	33.3	
Follow up (months), Mean (SD)		103.3 (65.5)		92.1 (48.7)		111.8 (76.8)		0.511^+^

^+^Student’s *t*-test; ^++^Fisher’s exact test.

### Surgical techniques

#### Midcarpal tenodeses

Midcarpal tenodeses were performed using three established techniques, each requiring a specific surgical approach based on tendon selection and the direction of tendon routing. All patients undergoing tenodesis had a scaphoidectomy and correction of the DISI deformity, with temporary stabilization achieved using K-wires until the reconstruction was completed.

The FCR over-capitate tenodesis was performed using a combined volar and dorsal approach, following the method originally described by Garcia-Elias and later refined by Luchetti et al. [10, 13, 14]. A distally based slip of the flexor carpi radialis tendon was harvested volarly and routed from the volar aspect of the wrist to the dorsal side through the midcarpal interval. After scaphoid excision and radiocarpal realignment, the tendon was passed over the neck of the capitate and looped around the radiotriquetral ligament to create a stabilizing sling. The dorsal limb was secured with non-absorbable sutures, and a dorsal capsular flap was advanced to reinforce the construct. This technique was selected in patients with preserved radiolunate and capitolunate cartilage who preferred a motion-preserving reconstruction.

The transosseous FCR “T-technique” tenodesis was also performed using a volar harvest of a distally based FCR tendon strip, but differed in its method of fixation. Consistent with previously described osseous-anchorage variations of FCR tenodesis [10, 13], a transosseous tunnel was created through the triquetrum from volar to dorsal, enabling the tendon to be delivered dorsally through bone. The free tendon end was then redirected proximally and anchored to the dorsal distal radius, producing a rigid “T-shaped” construct with strong mechanical stability. This technique was chosen when more robust correction of midcarpal malalignment was required compared with ligamentous methods.

The ECRB transosseous C–T tenodesis was performed using a double approach. A dorsal longitudinal incision was made over the 3rd extensor compartment at Lister’s tubercle, followed by a ligament-sparing capsulotomy. The capitate cartilage was inspected, and the scaphoid was excised. After preparation of the ECRB tendon flap, transosseous tunnels were created in the capitate and triquetrum (2.5–3.5 mm drill).Through an extended volar carpal tunnel incision, the ECRB flap was passed volarly through the capitate and dorsally through the triquetrum, then fixed under tension to the dorsal radius at Lister’s tubercle using a bone anchor.

#### Partial arthrodeses

**Figure 1:**
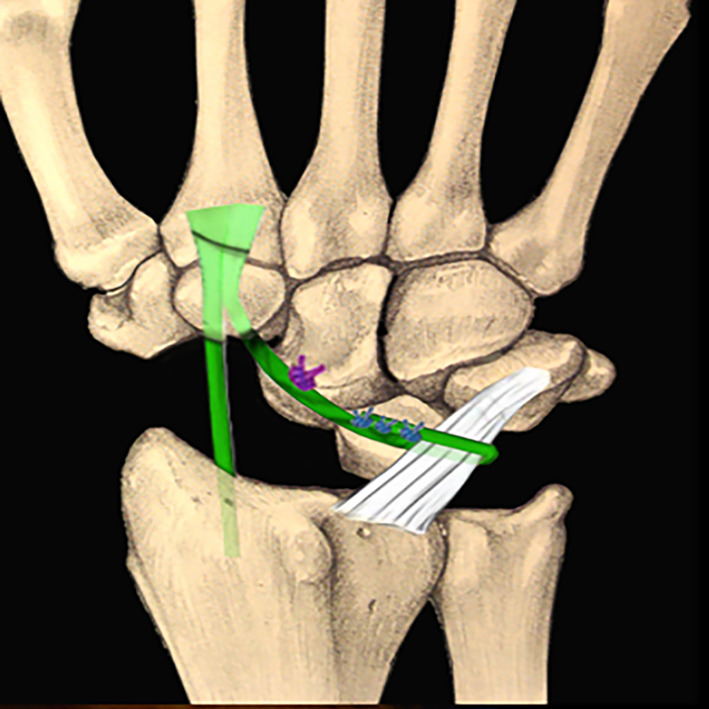
Scaphoidectomy and midcarpal tenodesis with the FCR tendon slip. The tendon is fixed at the neck of the capitate, passing around the Radiotriquetral ligament, and then sutured to itself (schematic). Source: Dr. Emmanouil Apergis.

**Figure 2:**
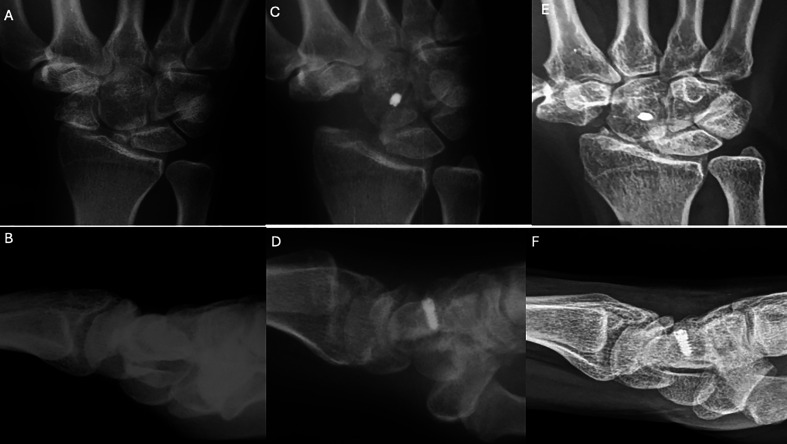
Clinical case of FCR tenodesis over capitate (Ligamentous grip) and Scaphoid excision. A and B. Preoperative wrist X-rays of SNAC stage II (AP and Lateral views). C and D. Postoperative wrist X-rays (AP and Lateral views) after the removal of K-wire (1month). E and F. Postoperative wrist X-rays (AP and Lateral views) after 12.25 years. Source: Dr. Emmanouil Apergis.

**Figure 3:**
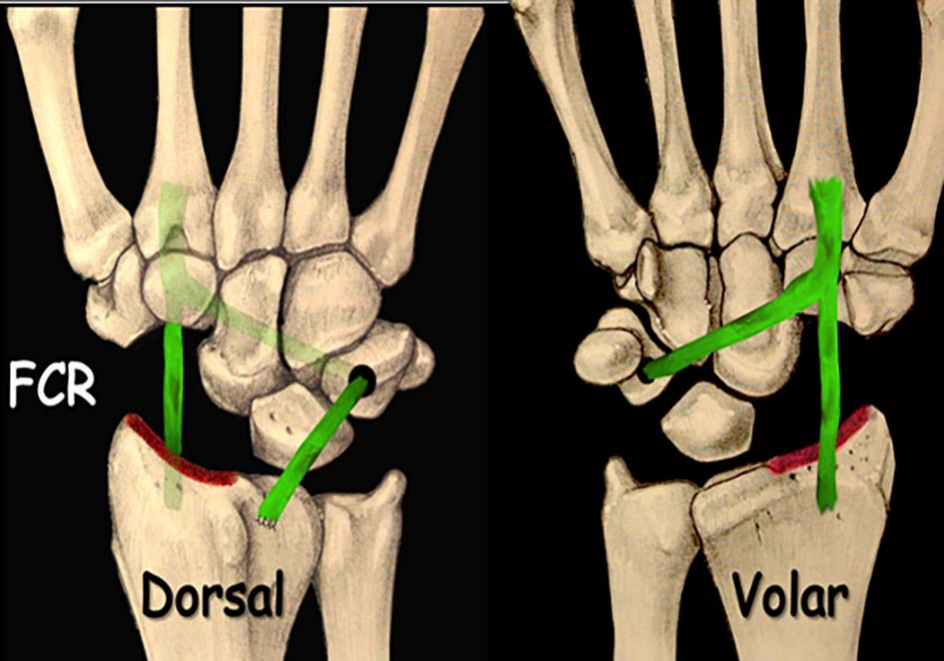
Scaphoidectomy and midcarpal tenodesis with the FCR transosseous T technique. (schematic). Source: Dr. Emmanouil Apergis.

**Figure 4:**
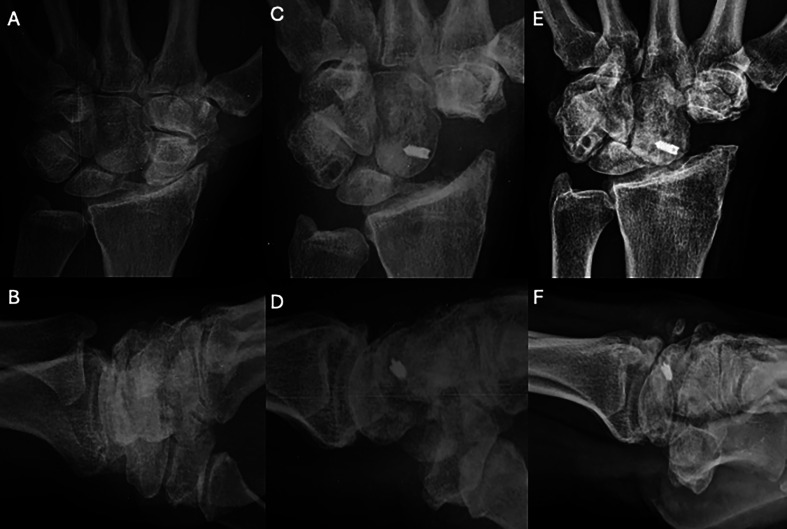
Clinical case of FCR Tenodesis transosseous T (Osseous grip) and Scaphoid excision. A and B. Preoperative wrist X-rays of SLAC stage II (AP and Lateral views). C and D. Postoperative wrist X-rays (AP and Lateral views) after 2 months. E and F. Postoperative wrist X-rays (AP and Lateral views) after 8.5 years. Source: Dr. Emmanouil Apergis.

**Figure 5:**
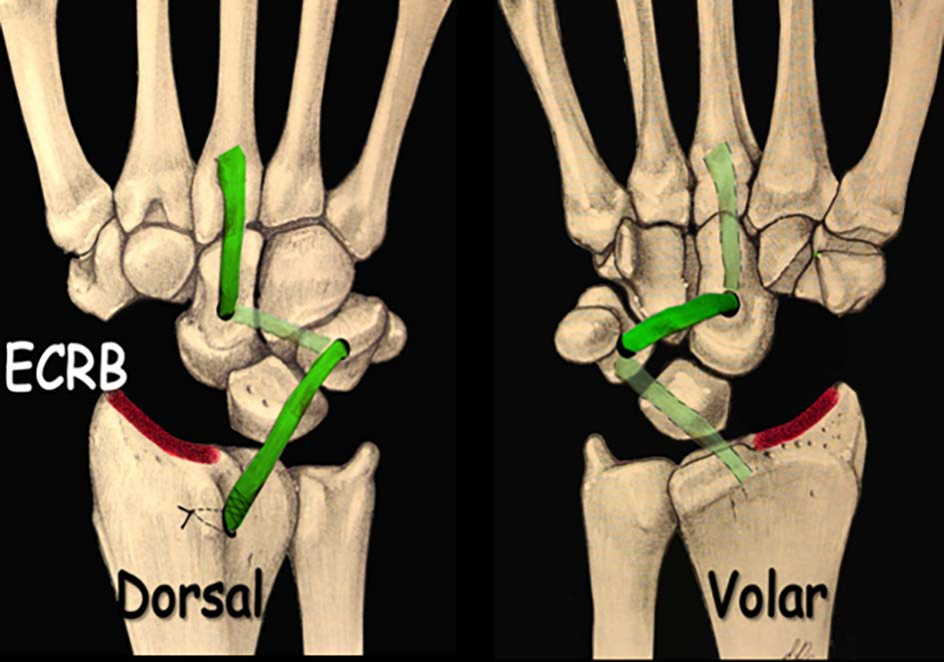
Scaphoidectomy and midcarpal tenodesis with the ECRB transosseous C-T technique. (schematic). Source: Dr. Emmanouil Apergis.

**Figure 6:**
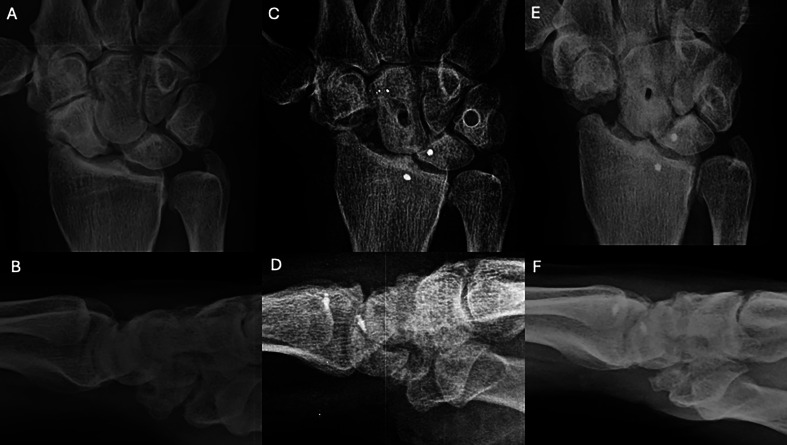
Clinical case of ECRB Transosseous C-T and Scaphoid excision. A and B. Preoperative wrist X-rays of SLAC stage II (AP and Lateral views). C and D. Postoperative wrist X-rays (AP and Lateral views) after 2 months. E and F. Postoperative wrist X-rays (AP and Lateral views) after 2 years. Source: Dr. Emmanouil Apergis.

**Figure 7:**
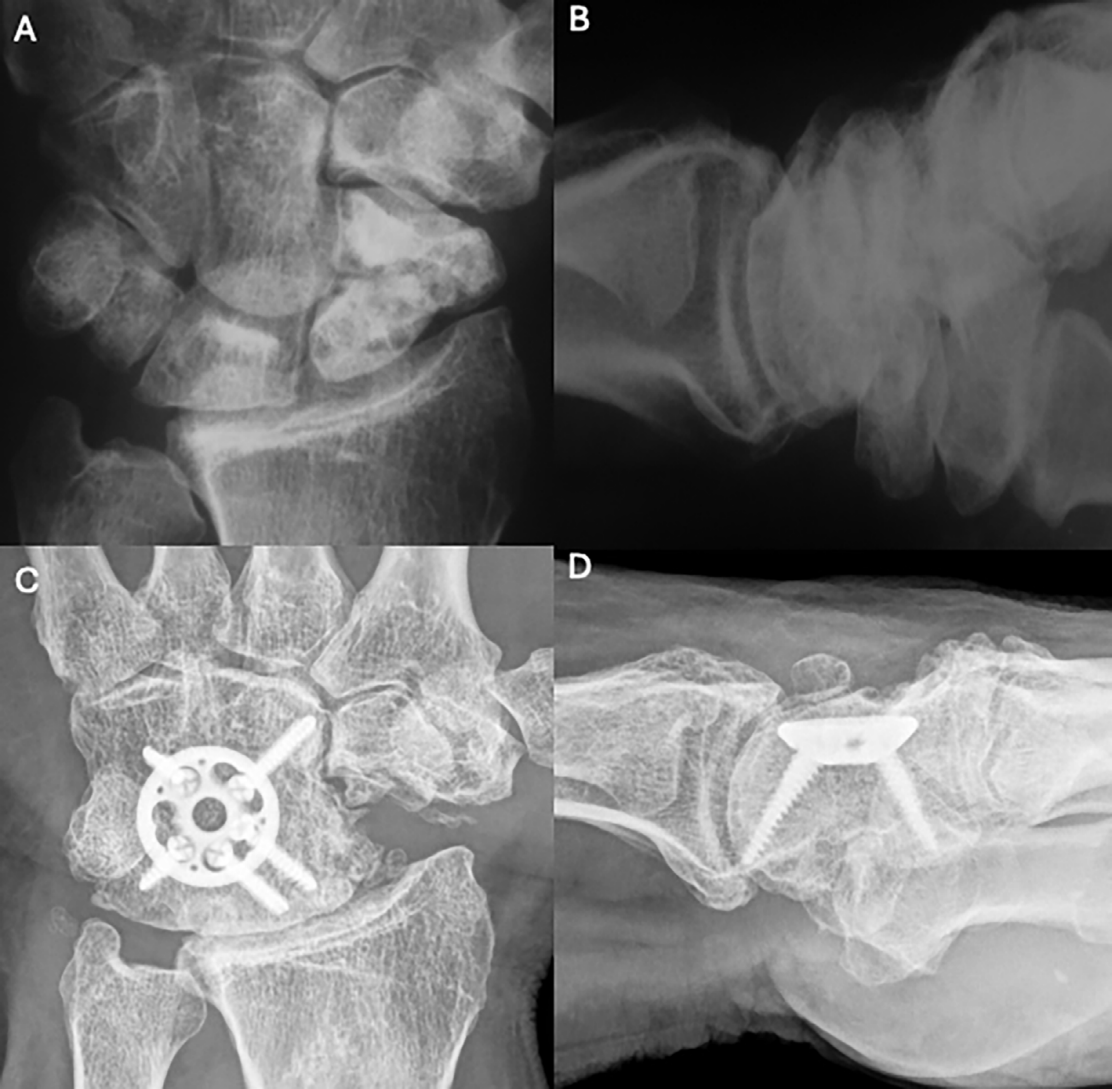
Clinical case of 4-Corner Fusion. A and B. Preoperative wrist X-rays of SNAC stage II (AP and Lateral views). C and D. Postoperative wrist X-rays (AP and Lateral views) after 17 years. Source: Dr. Emmanouil Apergis.

**Figure 8:**
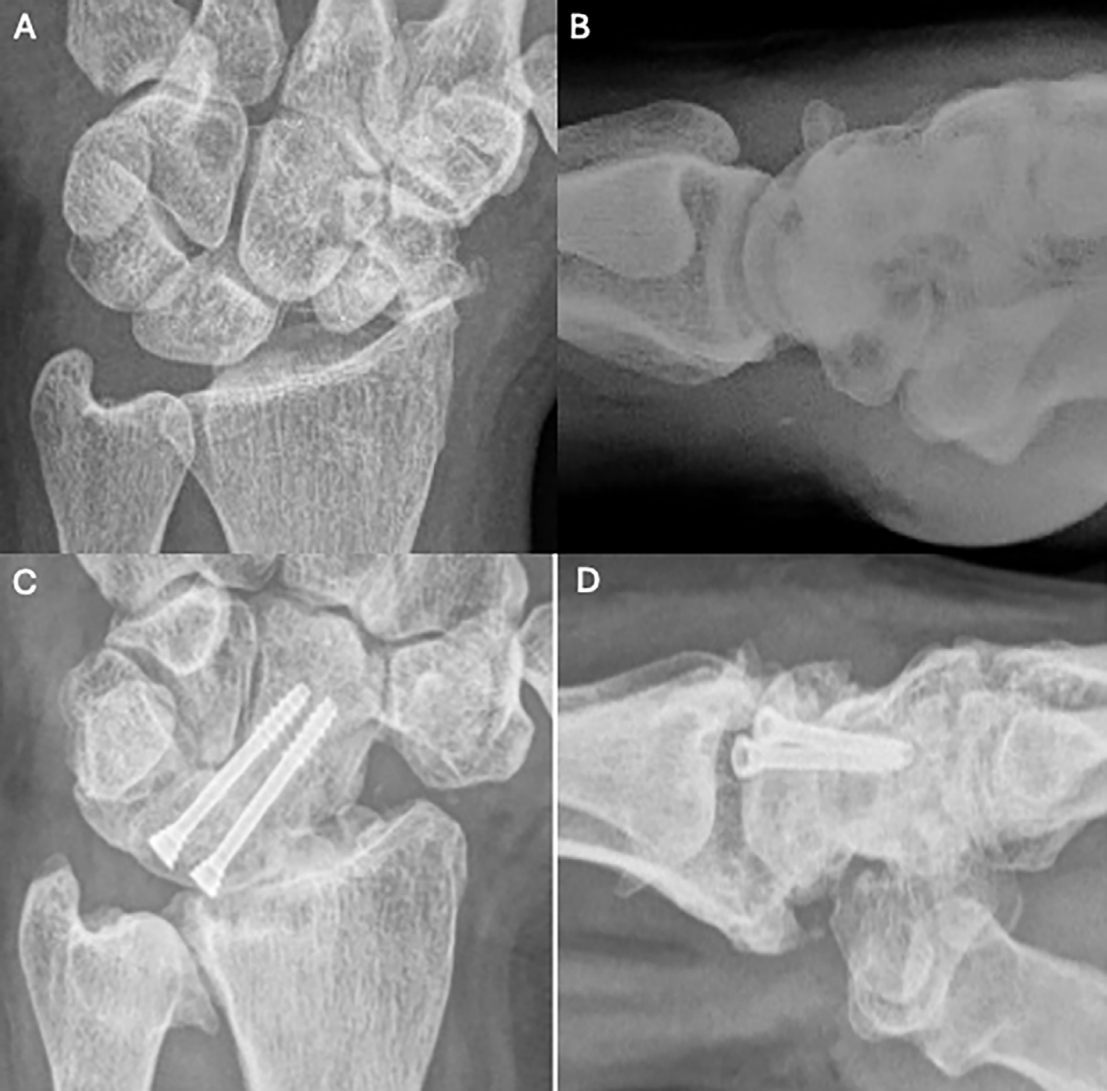
Clinical case of Capitolunate Fusion. A and B. Preoperative wrist X-rays of SNAC stage II (AP and Lateral views). C and D. Postoperative wrist X-rays (AP and Lateral views) after 4.7 years. Source: Dr. Emmanouil Apergis.

Partial arthrodeses (four-corner fusion and capitolunate fusion) were performed using standard dorsal approaches described in the classical carpal fusion literature [2–5, 15–20]. For four-corner fusion (4CF), the dorsal capsule was opened, the scaphoid excised, and the capitate, hamate, lunate, and triquetrum surfaces were prepared to bleeding bone. After provisional K-wire stabilization, definitive fixation was achieved using either a circular fusion plate or headless compression screws, supplemented with local autograft. For capitolunate fusion (CLF), only the lunate–capitate articulation was prepared before provisional reduction and fixation with headless compression screws, again with local bone graft as necessary. Both arthrodesis techniques have been widely validated in long-term series and systematic reviews, demonstrating high union rates and reliable clinical outcomes [3–5, 19, 20]. Illustrative schematics and representative clinical cases for all procedures are shown in Figures 1–8.

Postoperatively, all patients were immobilized in a volar wrist splint for six weeks before beginning gradual mobilization. Strengthening exercises were introduced between eight and ten weeks, and clinical and radiographic follow-up was conducted at standardized postoperative intervals.

## Statistical analysis

Data normality was tested with the Kolmogorov–Smirnov test. Continuous variables were compared using Student’s *t*-test or Mann–Whitney U test as appropriate; categorical variables were analyzed with Fisher’s exact test. A *p*-value < 0.05 was considered significant.

## Results

### Functional outcomes

Both treatment options resulted in comparable and durable functional outcomes. Mean postoperative grip strength was 27.9 ± 9.2 kg (~75% of the contralateral side), with no significant difference between tenodesis and arthrodesis. This range is consistent with previously reported postoperative strength following partial arthrodesis and motion-preserving techniques [3, 5]. Range of motion values – including extension, flexion, and radial/ulnar deviation – did not differ significantly between groups and were in line with established postoperative expectations for SLAC/SNAC salvage procedures [3, 5, 15]. Postoperative range of motion and grip strength values for each group are summarized in Table 2.

**Table 2:** Postoperative biometrics.

	Total	Type of surgery		*P*
		Tenodeses	Arthrodeses	
	Mean (SD)	Mean (SD)	Mean (SD)	
Grip	27.9 (9.2)	23.7 (8)	31 (9.1)	0.072^+^
Extension	34.5 (18)	35.1 (21.2)	34.1 (16.2)	0.901^+^
Flexion	41.4 (12.5)	43.1 (15.7)	40.2 (10)	0.606^+^
Ulnar deviation	29.3 (11.4)	29.2 (10.5)	29.4 (12.5)	0.970^+^
Radial deviation	15 (7.1)	17 (7.1)	13.6 (7)	0.283^+^

^+^Student’s *t*-test.

### Patient-reported outcomes

**Figure 9:**
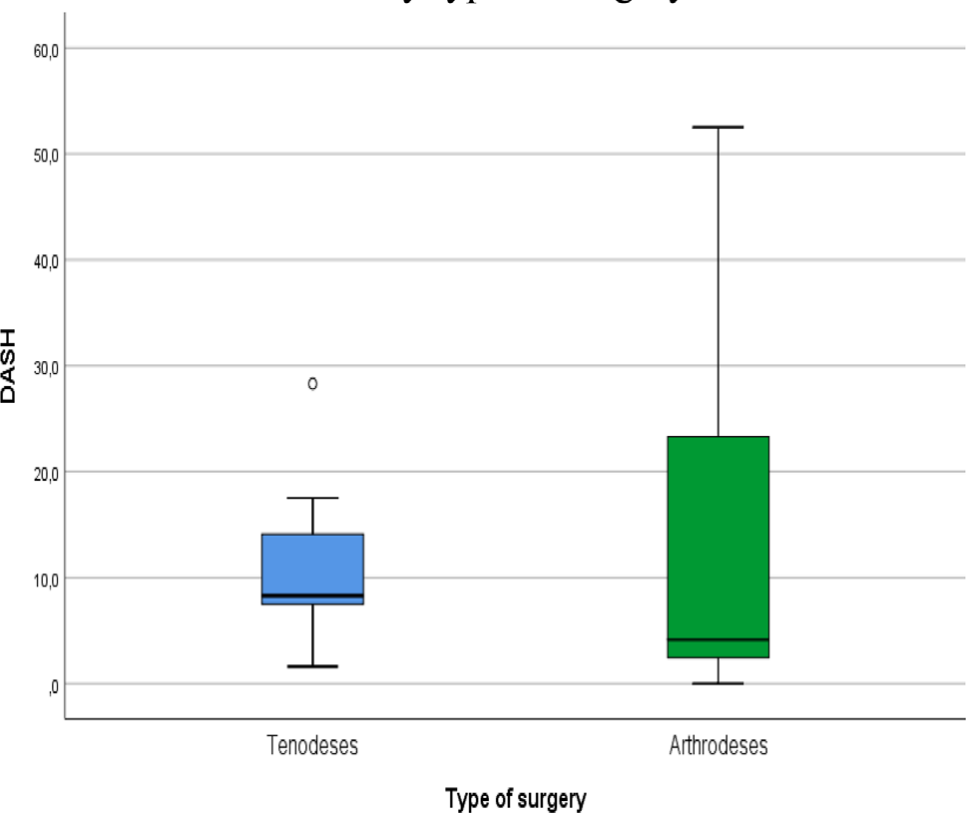
DASH scores by type of surgery.

**Figure 10:**
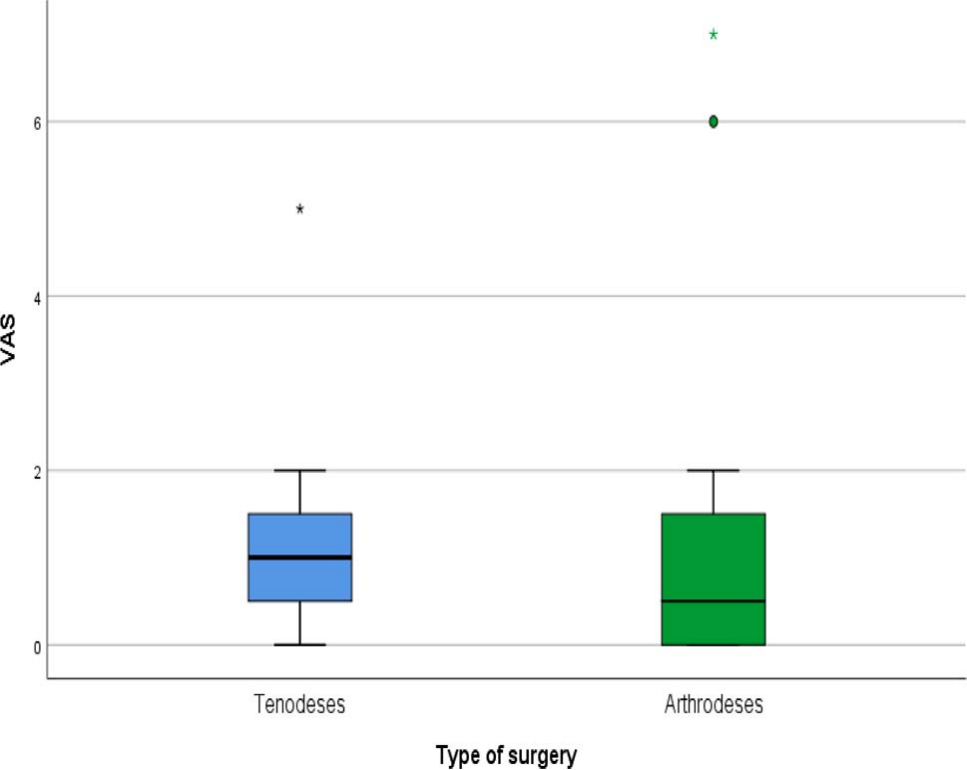
VAS scores by type of surgery.

**Figure 11:**
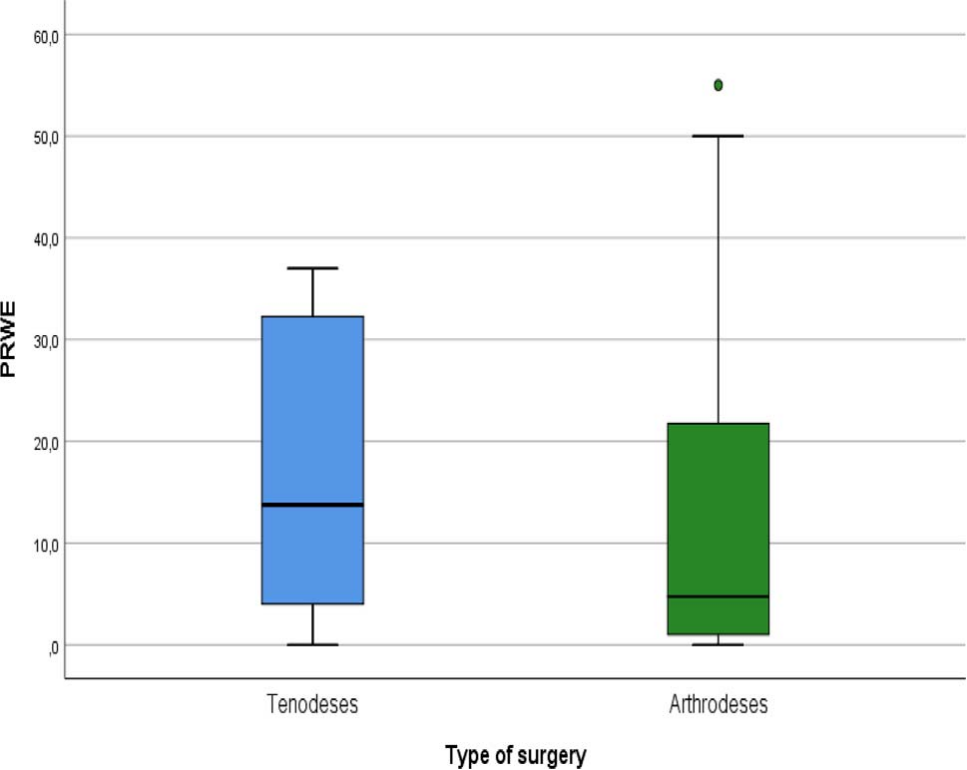
PRWE scores by type of surgery.

Patient-reported outcomes showed similarly favorable results. The mean DASH score (please see Figure 9) was 12.1 ± 14.2, with slightly lower scores in the tenodesis group, though without statistical significance. VAS pain scores were low (mean 1.5 ± 2.1) (please see Figure 10), and PRWE scores averaged 15.5 ± 17.6, indicating minimal residual symptoms (please see Figure 11). These PROM values correspond to those published in long-term follow-up of both 4CF/CLF and midcarpal tenodeses [3, 5, 11]. Patient-reported outcome measures (DASH, VAS, and PRWE) are summarized in Table 3.

**Table 3:** Postoperative scores.

	Total			Type of surgery					*P*
				Tenodeses			Arthrodeses		
	Mean (SD)	Median (IQR)	Mean (SD)		Median (IQR)	Mean (SD)		Median (IQR)	
DASH	12.1 (14.2)	7.5 (3.3–14.1)	10.9 (8.2)		8.3 (7.5–14.1)	13 (17.8)		4.2 (2.5–23.3)	0.422^+^
VAS	1.5 (2.1)	1 (0–2)	1.4 (1.7)		1 (0–2)	1.5 (2.4)		0.5 (0–1.5)	0.650^+^
PRWE	15.5 (17.6)	7 (2–27.8)	17.1 (15.2)		13.8 (4–32.3)	14.3 (19.6)		4.8 (1–21.8)	0.473^+^

^+^Mann–Whitney test.

### Radiographic outcomes

Radiographic outcomes demonstrated stable midcarpal alignment in all arthrodesis cases, with no evidence of non-union or hardware complications, consistent with the >90% union rate reported in large fusion cohorts and systematic reviews [3–5, 20]. In contrast, tenodesis patients demonstrated recurrent DISI deformity and widening of the triquetrocapitate interval during follow-up. However, no patient showed ulnar translocation of the lunate, and clinical symptoms did not correlate with radiographic progression – an observation also noted in prior tenodesis series [10, 13, 14].

### Complications and reoperations

No complications occurred in either group. Notably, no tenodesis patient required salvage arthrodesis, contrasting with earlier publications that report failure rates up to one-third [9, 10]. Similarly, the absence of complications in the arthrodesis group aligns with modern findings showing low reoperation rates when contemporary fixation methods are used [3, 4].

## Discussion

Scapholunate advanced collapse (SLAC) and scaphoid nonunion advanced collapse (SNAC) represent progressive patterns of degenerative wrist disease arising from chronic instability or nonunion, ultimately resulting in abnormal force transmission across the radiocarpal and midcarpal joints [1, 2]. Stage II disease occupies a critical point in this degenerative sequence: the radioscaphoid articulation is destroyed, but the radiolunate joint and, in many cases, the capitolunate joint maintain sufficient cartilage to allow for motion-preserving reconstructive procedures rather than obligatory arthrodesis [10, 11]. The present study demonstrates that midcarpal tenodeses and partial arthrodeses provide comparable long-term functional outcomes, with similar grip strength, range of motion, pain levels, and patient-reported outcome measures at a mean follow-up exceeding eight years. Because both midcarpal tenodeses and partial arthrodeses are reasonable options in this stage, treatment selection depends heavily on cartilage integrity, patient demands, and surgeon experience. However, despite the frequency with which stage II SLAC/SNAC is encountered, high-quality comparative studies remain scarce.

Our study has several limitations. The small sample size limits statistical power and reduces the ability to detect subtle differences between groups. Its retrospective design introduces selection bias, as procedure choice depended on intraoperative cartilage assessment and surgeon preference, although this reflects routine clinical practice for stage II SLAC/SNAC. All procedures were performed by a single surgeon, ensuring consistency but potentially limiting generalizability. Radiographic assessment, while standardized, may not capture subtle biomechanical changes. Similar observations have been reported in recent clinical series, where early capitolunate lesions were identified arthroscopically despite unremarkable radiographs [21]. These limitations are comparable to those reported in most SLAC/SNAC reconstruction series [3, 10]. Nonetheless, the long follow-up period remains a clear strength.

The absence of complications, non-unions, or reoperations in our series further supports the reliability of both methods when applied using careful selection criteria and consistent surgical technique. Our findings contribute directly to an area of the literature where comparative data are extremely limited. Key studies addressing surgical management of SLAC/SNAC are summarized in Table 4, allowing direct comparison with our results.

**Table 4:** Key studies comparison.

Author	Procedure(s)	Follow-up	Key findings
Watson and Ballet (1984) [2]	4CF	–	Defined SLAC progression; foundational technique
Wyrick et al. (1995) [15]	PRC vs 4CF	Mid-term	Comparable outcomes; PRC limited by capitate wear
Luchetti (2018) [10]	Tenodesis	5–9 yrs	Good clinical outcomes; radiographic deterioration
Nienstedt et al. (2023) [9]	Dynamic ECRB tenodesis	Long-term	Up to 33% required salvage fusion
Andronic et al. (2022) [3]	4CF (systematic review)	Long-term	>90% union; consistent PROMs
Traverso et al. (2017) [5]	4CF	≥10 yrs	Durable function; good patient satisfaction
Dunn et al. (2020) [4]	CLF (systematic review)	Long-term	Comparable results to 4CF
Elshahhat et al. (2024) [20]	CLF	Mid-term	PROMs comparable to 4CF
Reyniers et al. (2023) [11]	PRC vs 4CF	Long-term	Similar outcomes; PRC contraindicated with capitate chondrosis
Solgård et al. (2024) [12]	Limited intercarpal fusions	Mid-term	Comparable union; motion preserved

Our arthrodesis outcomes align closely with the robust literature supporting the reliability of both four-corner fusion (4CF) and capitolunate fusion (CLF). Systematic reviews and long-term studies consistently report union rates exceeding 90% and durable functional results [3–5, 20]. Traverso et al. documented excellent survivorship at ten years, with QuickDASH scores comparable to those observed in our cohort [5]. Recent clinical data have also shown favorable union rates and sustained functional improvement following capitolunate fusion in SNAC wrists, consistent with the stable radiographic and clinical outcomes noted in our arthrodesis group [22]. These findings continue to support partial arthrodeses as predictable and effective options for stage II SLAC/SNAC.

Outcomes following midcarpal tenodesis have historically been more variable. Earlier studies reported clinical improvement but frequent radiographic progression, leading to revision or conversion to arthrodesis in up to one-third of patients [9, 10, 13]. These findings contributed to skepticism regarding the durability of motion-preserving surgery. However, failures may be attributable to inadequate patient selection or unrecognized cartilage loss. In contrast, when cartilage is preserved – as in our cohort – long-term outcomes may be substantially better. None of our tenodesis patients required revision surgery, suggesting that precise indications and meticulous technique may mitigate the risks reported in earlier series.

Our data support the emerging viewpoint that midcarpal tenodeses, when applied appropriately, may offer sustained symptom relief comparable to arthrodeses while preserving mobility.

Radiographic outcomes differed significantly between groups. Arthrodesis patients maintained stable alignment and carpal height without signs of hardware compromise – an expected outcome consistent with fusion studies [3, 4]. Tenodesis patients often demonstrated recurrent DISI deformity and widening of the triquetrocapitate interval, mirroring earlier long-term reports [10, 13, 14]. Importantly, however, these radiographic changes did not correspond to deterioration in function or PROMs. This suggests that tendon-based dynamic reconstructions may maintain sufficient stability for functional use even without maintaining ideal radiographic alignment.

Accurate identification of cartilage status remains a major challenge in SLAC/SNAC management. Radiographs may significantly underestimate midcarpal degeneration, especially in the radiolunate or capitolunate joints [23]. Several authors advocate intraoperative inspection or arthroscopy to assess cartilage integrity more reliably [10, 24]. Early or subtle SL pathology is frequently misdiagnosed, leading to delayed intervention and progression to collapse [2, 11, 25]. In neglected scaphoid nonunion or chronic SL injury, the midcarpal joint may already be compromised, leaving arthrodesis as the more predictable option.

## Conclusion

Both partial arthrodeses and tenodeses provide satisfactory pain relief and functional improvement in stage II SLAC and SNAC wrists. Partial arthrodeses (4CF, CLF) offer durable outcomes with high union rates, but at the expense of motion and with the risk of hardware-related complications. Tenodeses (with FCR/ECRB rerouting) preserve motion and provide good functional outcomes, particularly in younger, high-demand patients with preserved cartilage surfaces, but are associated with a risk of radiographic progression and higher revision rates over time. Treatment should therefore be individualized. Tenodeses may be best suited for younger, active patients, prioritizing motion preservation, as long as the capitolunate cartilage is intact, accepting the possibility of a later salvage procedure. Partial arthrodeses are favored in older patients or those with midcarpal degeneration, where durable pain relief is the main goal.

Our findings support the role of tenodeses as an effective motion-preserving option for stage II SLAC/SNAC, while confirming the durability of partial arthrodeses. Ultimately, surgical decision-making must integrate patient age, cartilage status, and functional expectations.

## Data Availability

The authors confirm that all the data of this research are available upon request.
